# Feline leukemia virus point-of-care lateral flow tests have low positive predictive value in apparently healthy shelter cats

**DOI:** 10.3389/fvets.2026.1752228

**Published:** 2026-03-05

**Authors:** Hannah E. Urig, Kimberly A. Woodruff, W. Cooper Brookshire, David R. Smith

**Affiliations:** 1 Department of Pathobiology and Population Medicine, College of Veterinary Medicine, Mississippi State University, Starkville, MS, United States; 2 Department of Clinical Sciences, College of Veterinary Medicine, Mississippi State University, Starkville, MS, United States

**Keywords:** Bayesian latent class analysis, diagnostics, feline leukemia virus, predictive value, shelter

## Abstract

This study aimed to determine the true prevalence of feline leukemia virus (FeLV) infections in apparently healthy and sick shelter cats in Mississippi and estimate the predictive value of a lateral flow test results at the estimated true prevalences to guide testing recommendations. Blood samples (*n* = 383) were collected from a convenience sample of healthy and sick cats from February 2021 to July 2023. Blood serum samples from both apparently healthy and sick cats were tested for FeLV using lateral flow and insulated isothermal PCR (iiPCR) testing methods. Bayesian latent class modeling was used to estimate test performance and predictive value in both populations. The estimated true prevalence of FeLV in apparently healthy cats was 0.8% (95% CI 0.1%, 2.2%) and 5.3% (95% CI 1.3%, 11.5%) in sick cats. At these prevalences, the estimated positive predictive value of FeLV in healthy cats was 40.1% (95% CI 4.1%, 85.2%) and 99.0% (95% CI 99.4%, 100%) in sick cats. Negative predictive value of FeLV in healthy cats was 79.8% (95% CI 45.2%, 96.8%) and 99.2% (95% CI 97.1%, 100%) in sick cats. The predictive value of a positive test is low for healthy cats. Serial testing may not meaningfully improve the positive predictive value. Positive and negative predictive values were better for sick cats and may have diagnostic utility. Current testing methods may not be adequate for controlling the disease in population.

## 1 Introduction

Feline leukemia virus (FeLV) is an important infectious agent of cats causing immunodeficiency, an increased odds of developing opportunistic infections, and neoplasia (1).

FeLV is a single-stranded enveloped RNA virus. FeLV is classified into a number of subgroups, four of which are significant from a clinical standpoint. The virus can spread from one cat to another (horizontally) through a bite wound, during allogrooming, and rarely through the sharing of feeding dishes and litterboxes. Transmission from an infected queen to her nursing kittens (vertically), either prior to birth or during lactation, is also possible (2).

Following exposure, FeLV infection may result in four outcomes: abortive, regressive, progressive, or atypical (focal) infection (3, 4). The likelihood of each outcome is influenced by host and viral factors that shape the clinical course of disease. Approximately two-thirds of exposed cats develop abortive or regressive infection (5). Although the prognosis for FeLV-positive cats is guarded, persistently infected cats may survive for years with appropriate care (6, 7). Early infection is often subclinical; however, over time, cats may experience progressive health decline or recurrent illness. Clinical manifestations include weight loss, poor coat condition, lymphadenopathy, pale mucous membranes, recurrent infections, persistent fever, and ocular disease (3, 4).

Global apparent prevalence estimates range from 3 to 14% depending on geographic location, sex, lifestyle, and overall health status, and have declined in most countries, including the United States (2, 5, 7–10). The reported apparent prevalence of these infections in cats living in the United States ranges from 2% in healthy cats to 30% in high-risk or sick cats (3, 5–7, 11). In 2006, a survey of over 18,000 cats in the United States and Canada reported a 2.3% seroprevalence (2, 5, 7). A large study conducted in 2010, which evaluated cats from veterinary clinics and shelters in the USA and Canada, reported an apparent FeLV antigen prevalence of 3.1% (5). Although the prevalence of FeLV in shelter cats likely reflects the low rates found in pet cats, the number of infected cats to pass through shelters each year provides an opportunity for viral transmission prior to adoption that is unknown (2). Many shelter teams make euthanasia and adoption decisions based on commercially available FeLV point-of-care (POC) tests.

Although guidelines for feline retrovirus testing and management exist, there remains a need to improve recommendations due to the reported low prevalence of disease and the risk of a false positive outcome for cats in the shelter system. Current guidelines from the American Association of Feline Practitioners (AAFP) and the Association of Shelter Veterinarians (ASV), recommend that all cats eligible for adoption or relocation should be screened for retroviruses (4). However, based on the reported low prevalence rates and imperfect commercially available tests, testing of all cats in a shelter system may lead to an unnecessarily high risk of false positive test outcomes. Based on the testing recommendations and the currently available tests, this may lead to uninformed adoption and euthanasia decisions.

Effective management of FeLV depends on accurate diagnosis for both identifying infected cats and diagnosing retrovirus-associated disease. Several different diagnostic testing methods are available, such as quick lateral flow point- of care (POC) tests for antigen or antibody in plasma, virus isolation to find infectious viral particles in plasma, and a multitude of polymerase chain reaction (PCR) assays to find proviral DNA in the blood. The most frequently utilized tests in practice are immunoassays of various types. Speed and convenience are advantages of this form of testing, but there is little and inconsistent evidence available on how well they perform (2, 5, 12–14).

The lateral flow test is a commonly utilized POC test kit in shelters and is economically feasible for shelters. Published sensitivity and specificity values were 92.9% (95% CI 67.8%−98.8%) and 96.5% (95% CI: 82.1%−99.4%), respectively for FeLV antigen (15). It is important to note that other lateral flow testing method sensitivities and specificities may vary based on the brand of the product.

When a clear reference test or screening test is unavailable, alternative statistical methods have been established that allow for the evaluation of test sensitivity and specificity (16–20). One such method is based on Bayes' theorem, which incorporates error probabilities (prior distributions) derived from previous knowledge of the utilized tests, reference test, and prevalence into the analysis. By utilizing new likelihood data through a simulation known as Markov-chain Monte Carlo, the estimates of test accuracy and prevalence are adjusted (posterior distributions). This approach enables precise estimation of test accuracy even in situations where traditional methods would result in mistakes (19–22).

Performing BLCA becomes essential when examining two diagnostic tests across two populations with differing prevalence of infection in the absence of a gold standard test. BLCA's strengths lie in its ability to handle complex scenarios by accommodating uncertainties, incorporating prior knowledge, and accurately estimating parameters. In this context, where interactions between tests and populations can be complex, BLCA's flexibility allows for a more realistic representation of the underlying dynamics. By considering potential correlations between tests and accounting for imperfect reference standards, BLCA offers a nuanced approach that traditional methods often overlook. Furthermore, BLCA's simulation-based nature ensures robust estimates even with limited data, crucial when dealing with specific populations or rare conditions. Ultimately, BLCA empowers decision-making by providing comprehensive insights into the diagnostic performance of the tests across diverse populations, yielding results that are both accurate and informative for clinical, research, and public health applications.

The research question of this study was whether lateral flow tests commonly used in animal shelters provide clinically meaningful results given the low estimated prevalence of FeLV infection. Accordingly, the primary objective was to estimate the predictive value of lateral flow test results at the estimated true prevalence and to inform evidence-based testing recommendations. Due to financial limitations that restrict access to gold-standard diagnostics in shelter settings, a Bayesian latent class analysis was used.

## 2 Methods

### 2.1 Study design

A cross-sectional study was conducted to estimate the performance of a FeLV point-of-care lateral flow test [Zoetis Witness FeLV-FIV Rapid Test (ImmunoMigration)] by comparison with polymerase chain reaction [insulated isothermal PCR (iiPCR)].The study utilized BLCMs to compare two imperfect diagnostics tests applied to two populations with various disease prevalence. Bayesian latent class analysis (BLCA), a statistical approach based on a “non-criterion standard,” is employed to assess a range of tests for FeLV.

From February 2021 through July 2023, shelter cats were enrolled from two population groups, apparently healthy and clinically sick cats. Three hundred eighty-three (383), cats were recruited from five shelter locations across northern Mississippi at the time of admission. Due to cats entering shelter systems at variable times, samples were collected as intake examinations were performed. In total, 328 healthy cats (population 1) and 55 clinically sick cats (population 2) participated in the study. An apparently healthy cat was defined as having no obvious clinical signs of disease and a normal baseline physical examination. A clinically sick (clinically ill) cat was defined as having obvious clinical signs of disease on physical examination. Clinical signs of disease could include but were not limited to, ocular and nasal discharge coupled with coughing and or sneezing, diarrhea with the presence of blood and or mucous, vomiting, conjunctivitis, abscesses, and crackles and or wheezes on auscultation.

Cats had an unknown retroviral (FeLV or FIV) status at the time of enrollment. Only cats 6 months of age or greater were included in the study.

Blood was collected from each cat in compliance with the Mississippi State University Animal Care and Use Committee protocol 20–432 and 21–464. Blood samples were allowed to clot, centrifuged, and serum was collected. Each fresh serum sample was tested utilizing the lateral flow test and the remaining serum was frozen to −20 °C for iiPCR batch testing at a later time. Only cats for which there was sufficient sample volume to perform all tests were included in the study. Sera were tested using both rapid immunomigration and iiPCR according to the manufacturer's instructions.

#### 2.1.1 Testing methods

The lateral flow test used in this study was chosen because it is a commonly utilized POC test kit in shelters and its economic feasibility for shelters.

Samples were tested using a lateral flow test kit which was performed in accordance with the manufacturer's instructions. Positive results were recorded if two bands were present in the reading window for each respective virus. iiPCR was used as a comparative diagnostic tool for the evaluation of FeLV in each collected sample specimen. The iiPCR method used in the study is approved for use in research only and has not been licensed for commercial use by the United States Department of Agriculture Animal Plant Health Inspection Service (USDA APHIS) Center for Veterinary Biologics (CVB). For iiPCR testing, DNA was extracted from 200 μL of serum by using the taco™ mini preloaded DNA/RNA extraction set in accordance with the manufacturer's instructions (23). A nucleic acid extraction system (taco™ mini Automatic Nucleic Acid Extraction System, GeneReach Biotechnology Corporation, Taichung, Taiwan) was utilized for nucleic acid extraction. Insulated isothermal polymerase chain reaction (iiPCR) was performed by using a nucleic acid analyzer (POCKET™ Nucleic Acid Analyzer, GeneReach Biotechnology Corporation, Taichung, Taiwan) and FeLV reagent set with primers and probe designed to generate a fluorescent signal during amplification of target RNA (23). The primers and probe targeted the long terminal repeat (LTR) for FeLV and does not cross-react with nucleic acid from host and other feline pathogens (23).

The iiPCR reaction consisted of a designated FeLV reagent set (POCKET™ FeLV reagent set, GeneReach Biotechnology Corporation), which included a premix pack (lyophilized pellet, containing dNTPs, primers, probe, and enzyme for amplification); 50 μL of premix Buffer A (reaction buffer to re-dissolve the lyophilized pellet); 5 μL nucleic acid extract; and 5 μL positive [P(+)] control (reconstituted plasmid containing FeLV partial sequence as positive control). The reaction program was run for 60 min prior to the results being shown (23). Fluorescence was detected at 520 nm. Samples were deemed positive, negative, or questionable (repeat reaction with freshly prepared nucleic acid) on the monitor. The detection limit of the nucleic acid analyzer (POCKET™ Nucleic Acid Analyzer, GeneReach Biotechnology Corporation) FeLV reagent sets is about 10 copies per reaction (23).

### 2.2 Statistical analysis

#### 2.2.1 Latent class model

Results were tabulated using a computer spreadsheet (24). Test sensitivity and specificity were estimated by BLCA using statistical software (25). The model used a two-test-in-two population model, with different disease prevalence. It was assumed that the prevalence varied from one population (apparently healthy cats vs. clinically sick cats) to the other and the test sensitivity and specificity were held constant across the populations. It was anticipated that the latter population (clinically sick cats) would have a higher prevalence than the healthy cat population. As described by Hui and Walter (26), this would be advantageous to model the different populations simultaneously in the same BLCM (27).

#### 2.2.2 Model parameters

The model parameters were: (i) sensitivity and specificity of the lateral flow test kit, (ii) estimated sensitivity and specificity of iiPCR, and (iii) apparent prevalence for each of the two populations. The model is fully described in Supplementary material.

Positive (PPV) and negative predictive values (NPV) of each testing approach were computed for population 1 (apparently healthy cats) and 2 (clinically sick cats) according to Equations 1, 2 (16).


$$\begin{array}{r} Positive\;Predictive\;Value\;\left(PPV\right)=p{\left(D+\right)}^{*}Se/p{\left(D+\right)}^{*}Se+\\ {\left(1-p\left(D+\right)\right)}^{*}\left(1-Sp\right) \end{array}$$ (1)

$$\begin{array}{r} Negative\;Predictive\;Value\;\left(NPV\right)={\left(1-p\left(D+\right)\right)}^{*}Sp/\\ {\left(1-p\left(D+\right)\right)}^{*}Sp+p{\left(D+\right)}^{*}\left(1-Se\right) \end{array}$$ (2)

Where Se and Sp represent the sensitivity and specificity of the lateral flow test kit, respectively, and p(D+) is the estimated true prevalence in a given population.

Three Markov chains (MCs), ran for 101,000 iterations with a burn-in period of 1,000 iterations, were used to obtain posterior inferences (median and 95% Bayesian credible ranges). Visual inspection of the MCs' history plots was used to gauge the convergence of the MCs, and autocorrelation plots were examined to gauge the behavior of the chains.

#### 2.2.3 Prior information on test performance

Prior information about test sensitivity and specificity and disease prevalence were specified using beta distributions. These were calculated using statistical software (25, 28) and estimates of the test performance and disease prevalence provided by the lateral flow test kit manufacturer (5, 7, 15). The iiPCR test did not have estimated sensitivity and specificity reported, therefore, the values used for priors were estimated based on other reported PCR testing methodologies. Prevalence of disease was estimated based on prior published reports (2, 5, 7).

#### 2.2.4 Modeling

A Markov Chain Monte Carlo (MCMC) simulation was used to estimate the median and 95% credible intervals from the posterior distributions. For each analysis, an initial burn in 1,000 iterations was discarded, and mode estimates were based on a further 101,000 iterations. Convergence for each model was assessed by simultaneously running 3 chains with widely differing starting values.

The models were run with different priors to assess the effect of prior knowledge on estimations of diagnostic performance. As recommended by Johnson et al., and Dufour and Arango Sabogal, the percentiles were set to be more diffuse by 30% and the mode for sensitivity and specificity of each individual lateral flow test kit (diagnostic screening test) were perturbed by 10% for the sensitivity analysis (Table 1) (27, 29).

**Table 1:** Prior distributions used for unknown parameters for two models.

**FeLV parameters**	**Dependence elicited priors (mode)**	**dBeta**	**Alternative perturbed priors (mode)**	**dBeta**
Prevalence Pop1 (healthy cats)	0.01	42.778	0.01	42.778
Prevalence Pop2 (sick cats)	0.035	36.76014	0.035	36.76014
PCR Se	0.95	1.261421	0.95	1.261421
PCR Sp	0.9	1.649333	0.9	1.649333
Lateral flow test Se (FeLV)	0.929	1.975581	0.829	1.83953
Lateral flow test Sp (FeLV)	0.965	1.957912	0.865	2.160688

Prior distributions used for the unknown parameters for two models: (1) a model allowing for conditional dependence between tests and with prior distribution elicited from the scientific literature (dependence); (2) a similar model where the elicited priors were perturbed i.e., mode was modified and/or variance was increased (Alternative).

#### 2.2.5 Number needed to treat

Number needed to treat (NNT) was computed for population 1 (apparently healthy cats) and 2 (clinically sick cats) according to Equation 6 (16).


$$\begin{array}{l} Experimental\;Event\;Rate\;\left(EER\right)=\left(\frac{A}{A+B}\right) \end{array}$$ (3)

$$\begin{array}{l} Control\;Event\;Rate\;\left(CER\right)=\left(\frac{C}{C+D}\right) \end{array}$$ (4)

$$\begin{array}{l} Absolute\;Risk\;Reduction\;\left(ARR\right)=|CER-EER| \end{array}$$ (5)

$$\begin{array}{l} Number\;Needed\;to\;Treat=\;1/ARR \end{array}$$ (6)

Where absolute risk reduction (ARR) is represented as the difference in risk between the treated and control groups.

## 3 Results

Based on the overall sample population of apparently healthy cats, the median age was 24 months, while the median age was 36 months for clinically sick cats.

### 3.1 Feline leukemia virus prevalences

**Figure 1:**
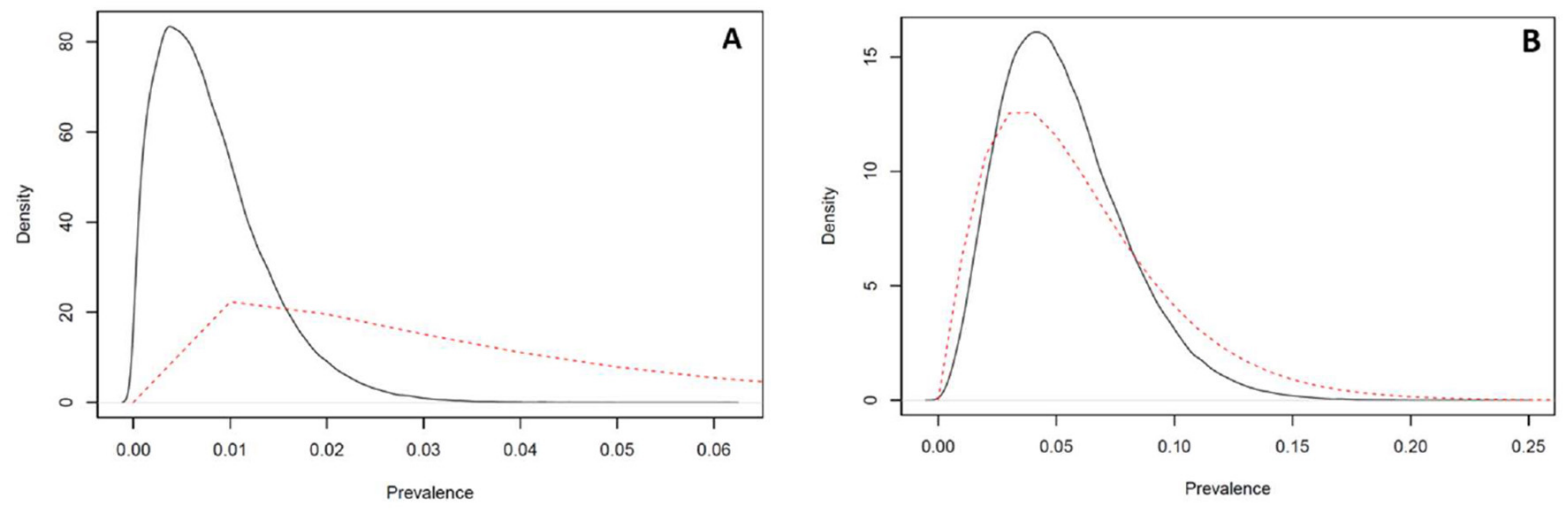
Estimated true prevalence of feline leukemia virus (FeLV) in Mississippi shelter cat populations. **(A)** The estimated true prevalence of FeLV in the apparently healthy cat population was 0.8% and 95% credible interval of 0.1%−2.2%. **(B)** The estimated true prevalence distribution of FeLV in the clinically sick cat population was 5.3% and 95% credible interval of 1.3%−11.5%. **(A, B)** The red dashed line indicates the model priors in the Bayesian model, and the solid black line indicates the model estimated true prevalence of disease in the respective populations.

The test results obtained for the 328 apparently healthy cats and 55 clinically sick shelter cat samples are summarized in Table 2. An estimated true prevalence of feline leukemia virus within the apparently healthy cat sampled population was 0.8% (0.1%−2.2%) (Figure 1) and 5.3% (1.3%−11.5%) in the clinically sick cat sampled population (Figure 1).

**Table 2:** Number of positive (+), negative (–) results by iiPCR and lateral flow test (diagnostic screening) tests for FeLV and FIV.

	**Test Result**	Apparently healthy cats		Clinically sick cats	
FeLV		**PCR** +	**PCR –**	**PCR** +	**PCR –**
	Lateral flow test +	2	1	2	0
	Lateral flow test –	3	322	3	50

### 3.2 FeLV diagnostic test performance and predictive values

**Figure 2:**
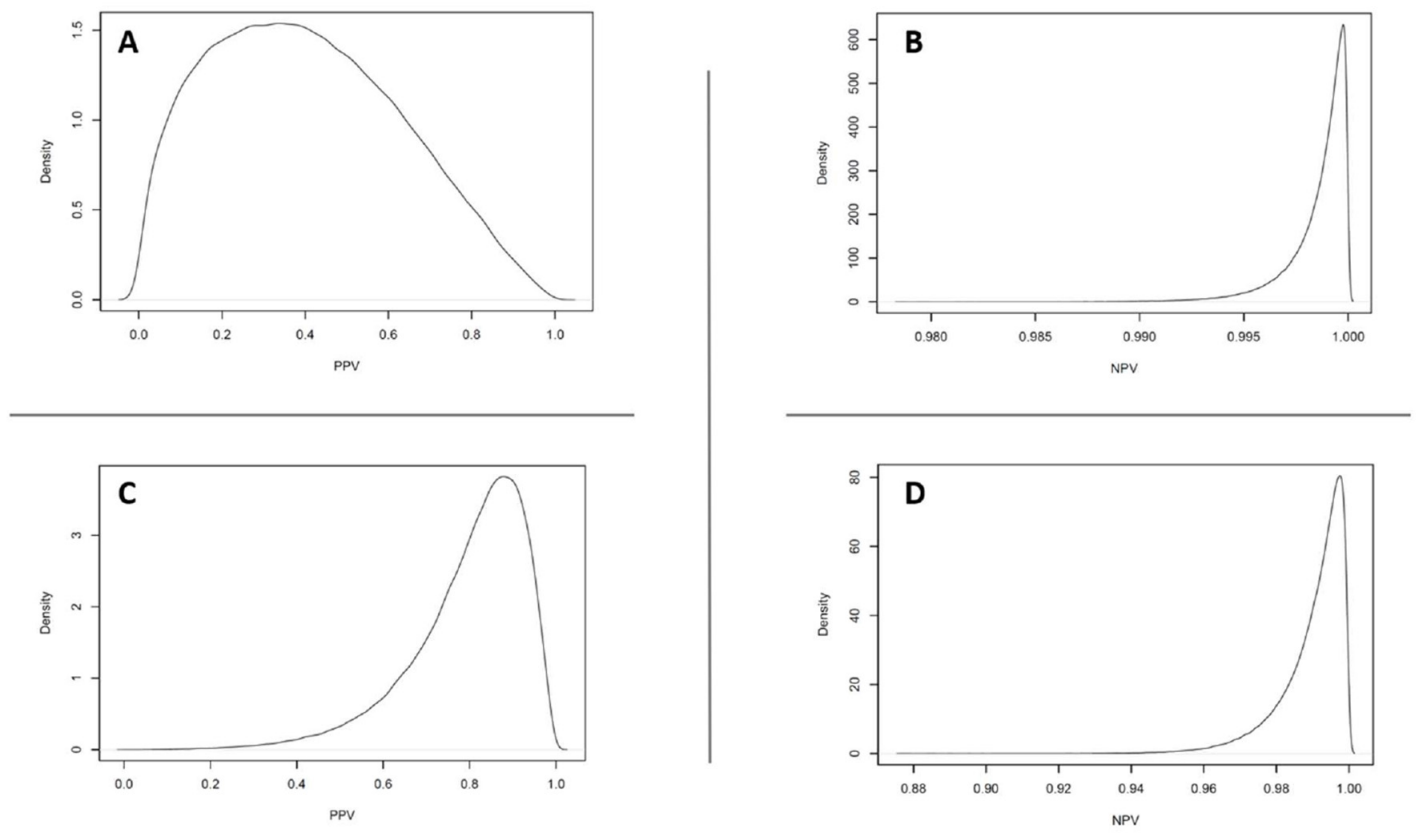
Estimated predictive value distributions for FeLV using a point-of-care lateral flow test in Mississippi shelter cat populations. **(A)** The estimated positive predictive distribution for a lateral flow test in apparently healthy (Prev 1) shelter cat populations for FeLV was 40.1% and 95% credible interval of 4.1%−85.2%. **(B)** The estimated negative predictive distribution in apparently healthy (Prev 1) Mississippi shelter cat populations for FeLV was 99.9% and 95% credible interval of 99.4%−100%. **(C)** The estimated positive predictive distribution for a lateral flow test in clinically sick (Prev 2) shelter cat populations was 79.8% and 95% credible interval of 45.2%−96.8%. **(D)** The estimated negative predictive distribution in clinically sick (Prev 2) Mississippi shelter cat populations for FeLV was 99.0% and 95% credible interval of 96.6%−99.9%.

Mean sensitivity estimates for the lateral flow and PCR tests were 83.4% (61.1%−97.7%) and 86.1% (59.1%−99.2%), respectively. Mean specificity estimates for the lateral flow and iiPCR tests were 99.0% (97.6%−99.8%) and 97.8% (95.9%−99.2%), respectively. The corresponding mean positive predictive value (PPV) and negative predictive value (NPV) in the healthy cat population for the lateral flow test were 40.2% (4.1%−85.0%) (Figure 2) and 99.9% (99.4%−100%) (Figure 2), respectively. The mean PPV and NPV in the clinically sick cat population for the lateral flow test were 79.5% (45.2%−96.8%) (Figure 2) and 99.0% (96.5%−99.9 %) (Figure 2), respectively. Mean PPV and NPV in the apparently healthy cat population for the iiPCR test were 24.5% (2.3%−62.4%) and 99.9% (99.5%−100%), respectively. The mean PPV and NPV in the clinically sick cat population for the iiPCR test were 65.5% (28.2%−91.0%) and 99.2% (97.1%−100%), respectively.

The posterior distributions of the models with perturbed priors (alternative model) and the model assuming conditional dependence (main model) are presented in Table 3 for the FeLV model. The alternative model results suggested that the diagnostic accuracy of lateral flow test and iiPCR did not depend highly on the prior information, since no major differences were observed between the alternative model and the main model.

**Table 3:** Mean posterior estimates (95% Bayesian credibility interval) and covariance terms (CovP, CovN) obtained for FeLV prevalence in two populations of shelter cats (Populations 1: apparently healthy cats; Population 2: clinically sick cats).

**Parameter**	**Main: conditional dependence**	**Alternative: perturbed priors**
Prevalence in population 1 (healthy)	0.8 (0.1–2.2)	0.8 (0.1–2.3)
Prevalence in population 2 (sick)	5.3 (1.3–11.5)	5.2 (1.2–11.4)
Se of PCR	86.1 (59.1–99.2)	77.4 (38.9–98.6)
Se of lateral flow test	83.4 (61.1–97.7)	83.6 (61.2–97.7)
Se of parallel interpretation	95.5 (83.2–99.9)	94.4 (79.8–99.9)
Se of series interpretation	74.0 (45.7–93.3)	66.7 (29.4–91.5)
Sp of PCR	97.8 (95.9–99.2)	97.8 (95.9–99.3)
Sp of lateral flow test	99.0 (97.6–99.8)	99.0 (97.6–99.8)
Sp of parallel interpretation	97.3 (95.4–98.9)	97.4 (95.4–98.9)
Sp of series interpretation	99.4 (98.3–100)	99.4 (98.3–100)
PPV of PCR in population 1 (healthy)	24.5 (2.3–62.4)	23.1 (1.9–61.4)
PPV of lateral flow test in population 1 (healthy)	40.1 (4.1–85.2)	40.9 (4.3–86.0)
PPV of parallel interpretation in population 1 (healthy)	22.8 (2.2–56.4)	23.0 (2.2–57.0)
PPV of series interpretation in population 1 (healthy)	52.2 (5.4–97.4)	49.9 (4.4–97.1)
NPV of PCR in population 1 (healthy)	99.9 (99.5–100)	99.8 (99.2–100)
NPV of lateral flow test in population 1 (healthy)	99.9 (99.4–100)	99.9 (99.4–100)
NPV of parallel interpretation in population 1 (healthy)	100 (99.8–100)	99.9 (99.7–100)
NPV of series interpretation in population 1 (healthy)	99.8 (99.2–100)	99.7 (99.0–100)
PPV of PCR in population 2 (sick)	65.5 (28.2–91.0)	62.2 (22.1–90.6)
PPV of lateral flow test in population 2 (sick)	79.8 (45.2–96.8)	79.2 (43.9–96.9)
PPV of parallel interpretation in population 2 (sick)	63.9 (28.5–88.3)	63.1 (27.2–88.3)
PPV of series interpretation in population 2 (sick)	85.6 (50.1–99.5)	83.1 (40.4–99.5)
NPV of PCR in population 2 (sick)	99.2 (97.1–100)	98.8 (95.9–99.9)
NPV of lateral flow test in population 2 (sick)	99.0 (96.6–99.9)	99.0 (96.6–99.9)
NPV of parallel interpretation in population 2 (sick)	99.7 (98.8–100)	99.7 (98.5–100)
NPV of series interpretation in population 2 (sick)	98.5 (95.5–99.8)	98.2 (94.6–99.8)
CovP	2.2 (−3.5–10.9)	2.0 (−5.9–11.9)
CovN	0.5 (0.0–1.7)	0.6 (0.0–1.7)

## 4 Discussion

To our knowledge, this is the first study to evaluate this commercial lateral flow POC test and serial iiPCR testing methods using BLCA, although a previous study also used BLCA modeling to evaluate other FeLV diagnostic lateral flow tests (19). Even though BLCA modeling is well-established in the field of veterinary epidemiology, it has only been utilized in a small sample of evaluations of veterinary diagnostic tests (19, 30). The BLCA model used in the current study was modeled to allow for the evaluation of two independent tests by using sampled data from two populations (19, 29, 31).

Bayesian modeling stands out for its ability to consider uncertainty in test performance and disease prevalence when analyzing data. This approach enables estimation of test sensitivity and specificity even when true disease status is unknown, making it particularly valuable when reference tests are imperfect or when disease stage influences test accuracy (17, 19, 21, 22).

This study estimated the true prevalence of FeLV in healthy and clinically sick shelter cat populations. Previous studies in 2006 and 2010 reported an apparent prevalence of 2.3–3.1% FeLV, but these studies evaluated cats from veterinary clinics and shelters in the United States and Canada and did not adjust for health status or diagnostic error (4, 5, 7). Consideration of diagnostic error is essential, as our analysis shows that the expected risk of false-positive results is similar to the reported prevalence in many studies. It is also important to have an estimate of true prevalence in healthy and clinically sick cats to accurately estimate pre-test probability and ultimately positive and negative predictive values in those populations.

Another objective of this study was to assess the predictive values for the lateral flow test and the iiPCR. The PPV for FeLV had a wide 95% credible interval which indicates a large degree of uncertainty about the predictive value of a positive test result. Even so, the expected value suggests that 60% of healthy cats that test positive for FeLV are misclassified. False positive results may occur due to low test specificity in a low disease prevalence population. and leading to adverse outcomes, such as euthanasia, decreased adoptability, or housing with truly infected animals, resulting in an increased risk true infection.

The necessity of testing all cats for retroviral infection prior to adoption from shelters has been taught and implemented for years, however, due to the relatively low positive predictive value, this may not be the best advice for healthy appearing cats depending on several factors related to disposition of test-positive cats and willingness to invest in multiple testing. Shelters across the country may rely on various point-of-care (POC) tests, such as the lateral flow test (used in the current study), to determine the FeLV infection status of cats entering their populations. Based on the 0.8% estimated true prevalence of the sample population for FeLV in apparently healthy cats, the predictive value of a positive test result in this population is low. Apparently healthy cats that test positive on POC tests are more likely to be false positives. On the other hand, a positive test result increases the suspicion that the cat is FeLV infected from very low (0.8%) pre-test probability to something much higher (40%) post-test probability. In this case, serial testing positive-testing cats with a second, highly specific test might improve diagnostic precision. However, in this study, serial testing with iiPCR only yielded a slightly higher positive predictive value (Table 3).

The NPV in apparently healthy cats was high; however, the true prevalence of infection in test negative healthy cats was not meaningfully different from the true prevalence prior to testing. Therefore, a negative test result does greatly modify our beliefs about the FeLV status of an apparently healthy cat at the time of testing.

The expense and effort of testing to retain only test-negative cats for adoption yields little practical improvement in prevalence of infection (0.8% to 0.1%). The number needed to treat for use of the POC lateral flow test for FeLV screening of healthy cats would be 143. So, if testing a healthy appearing cat cost $15.00, the cost to remove each FeLV-infected cat would be $2,145.

Among clinically sick cats entering the shelter, the mean PPV for FeLV was 80% and NPV was 99%. Therefore, both a positive and negative lateral flow test result from a clinically sick cat admitted into the shelter would have reasonable likelihood to present the true infection status of the animal. A positive test result would be more believable and decisions regarding the outcomes for test-positive cats could be made with more confidence of the true infection status of the animal. While a negative test result on a sick cat should be re-evaluated in 60 days to obtain a reliable test result. Therefore, testing clinically sick cats at admission to a shelter may be reliable and that information might be useful to manage the biocontainment of FeLV in the shelter and subsequently adopted populations of cats. The number needed to treat when testing clinically sick cats is approximately twenty, so, for example, if testing sick cats' cost $15, the cost to remove each FeLV-infected cat would be $300.

In a shelter setting, a relatively small sample size may be adequate for FeLV testing because the primary objective is often population-level screening rather than precise estimation of individual-level prevalence. Given the low expected prevalence of FeLV in many shelter populations, larger sample sizes yield diminishing returns in improving predictive value estimates, while smaller samples can still provide meaningful information to guide testing strategies and resource allocation.

The sensitivity analysis conducted on the perturbed parameters as seen in Table 3, reveals the potential impact of parameter variations on our study outcomes. The results of the sensitivity analysis for FeLV fell within 1% of the original model results, except for the sensitivity of PCR, sensitivity of series interpretation, and positive predictive value of series interpretation in apparently healthy cats. This is indicative of a stable model.

## 5 Conclusion

The present study estimated the true prevalence of FeLV in apparently healthy cats and clinically sick shelter cats across Mississippi, the diagnostic test performance of the lateral flow test, and the predictive value of the tests in those respective populations. Based on the estimated true prevalence of disease in healthy cat populations entering shelter systems, the predictive value of a positive test for testing apparently healthy cats is low. Therefore, the authors believe healthy cats should not be screened unless there is willingness and resources to commit to serial testing with a highly specific follow-up test. On the other hand, testing clinically sick cats provides more reliable predictive value and may be helpful for making adoption decisions in shelter systems.

## Data Availability

The raw data supporting the conclusions of this article will be made available by the authors, without undue reservation.
