# Supplementary material for: Feline leukemia virus point-of-care lateral flow tests have low positive predictive value in apparently healthy shelter cats
Source: Front Vet Sci. 2026 Mar 5;13:1752228. doi: 10.3389/fvets.2026.1752228 (PMC12999380; doi:10.3389/fvets.2026.1752228)
Supplement: Supplementary file 1 [file Data_Sheet_1.docx]

Model used to estimate Zoetis Witness FeLV-FIV rapid test (screen) and iiPCR (PCR)

#POPULATION SENSITIVITY AND SPECIFICITY.

#PPV, NPV, AND DISEASE PREVALENCE IN APPARENTLY HEALTHY CATS (POPULATION 1) AND CLINICALLY ILL CATS (POPULATION 2) ENTERING SHELTERING SYSTEMS ACROSS NORTHERN MISSISSIPPI ASSUMING CONDITONAL DEPENDENCE BETWEEN THE SENSITIVITIES AND SPECIFITIES OF SCREENING AND iiPCR TESTS.

#====================================================================================================#.

Model{.

#=== LIKELIHOOD ===#

#=== POPULATION 1 ===#

Pop1[1:4] ~ dmulti(p1[1:4], ",nPop1,")

p1[1] <- Prev1*(Se_", TestA, "*Se_", TestB, " + covp) + (1-Prev1)*((1-Sp_", TestA, ")*(1-Sp_", TestB, ") + covn)

p1[2] <- Prev1*(Se_", TestA, "*(1-Se_", TestB, ") - covp) + (1-Prev1)*((1-Sp_", TestA, ")*Sp_", TestB, " - covn)

p1[3] <- Prev1*((1-Se_", TestA, ")*Se_", TestB, " - covp) + (1-Prev1)*(Sp_", TestA, "*(1-Sp_", TestB, ") - covn)

p1[4] <- Prev1*((1-Se_", TestA, ")*(1-Se_", TestB, ") + covp) + (1-Prev1)*(Sp_", TestA, "*Sp_", TestB, " + covn)

#=== POPULATION 2 ===#

Pop2[1:4] ~ dmulti(p2[1:4], ",nPop2,")

p2[1] <- Prev2*(Se_", TestA, "*Se_", TestB, " + covp) + (1-Prev2)*((1-Sp_", TestA, ")*(1-Sp_", TestB, ") + covn)

p2[2] <- Prev2*(Se_", TestA, "*(1-Se_", TestB, ") - covp) + (1-Prev2)*((1-Sp_", TestA, ")*Sp_", TestB, " - covn)

p2[3] <- Prev2*((1-Se_", TestA, ")*Se_", TestB, " - covp) + (1-Prev2)*(Sp_", TestA, "*(1-Sp_", TestB, ") - covn)

p2[4] <- Prev2*((1-Se_", TestA, ")*(1-Se_", TestB, ") + covp) + (1-Prev2)*(Sp_", TestA, "*Sp_", TestB, " + covn)

#=== PRIOR ===#

Prev1 ~ dbeta(",Prev1.shapea,", ",Prev1.shapeb,") ## Prior for Prevalence in population 1

Prev2 ~ dbeta(",Prev2.shapea,", ",Prev2.shapeb,") ## Prior for Prevalence in population 2

Se_", TestA, " ~ dbeta(",Se.TestA.shapea,", ",Se.TestA.shapeb,") ## Prior for Se of Test A

Sp_", TestA, " ~ dbeta(",Sp.TestA.shapea,", ",Sp.TestA.shapeb,") ## Prior for Sp of Test A

Se_", TestB, " ~ dbeta(",Se.TestB.shapea,", ",Se.TestB.shapeb,") ## Prior for Se of Test B

Sp_", TestB, " ~ dbeta(",Sp.TestB.shapea,", ",Sp.TestB.shapeb,") ## Prior for Sp of Test B

#=== CONDITIONAL DEPENDENCE STRUCTURE ===#

covp ~ dunif(minp,maxp)

covn ~ dunif(minn,maxn)

minp <- (1-Se_", TestA, ")*(Se_", TestB, "-1)

minn <- (Sp_", TestA, "-1)*(1-Sp_", TestB, ")

maxp <- min(Se_", TestA, ",Se_", TestB, ") - Se_", TestA, "*Se_", TestB, "

maxn <- min(Sp_", TestA, ",Sp_", TestB, ") - Sp_", TestA, "*Sp_", TestB, "

#=== COMPUTING PPV AND NPP ===#

PPV_PCR_Prev1 <- (Prev1 *Se_PCR) / (((1-Prev1)* (1-Sp_PCR)) + (Prev1 *Se_PCR))

NPV_PCR_Prev1 <- (Sp_PCR *(1-Prev1)) / ((Sp_PCR*(1-Prev1)) + (Prev1 *(1-Se_PCR)))

PPV_Screen_Prev1 <- (Prev1 *Se_Screen) / (((1-Prev1)*(1-Sp_Screen)) + (Prev1 *Se_Screen))

NPV_Screen_Prev1 <- (Sp_Screen *(1-Prev1))/((Sp_Screen *(1-Prev1)) + (Prev1 *(1-Se_Screen)))

PPV_PCR_Prev2 <- (Prev2*Se_PCR) / (((1-Prev2)*(1-Sp_PCR)) + (Prev2 *Se_PCR))

NPV_PCR_Prev2 <- (Sp_PCR*(1-Prev2)) / ((Sp_PCR*(1-Prev2)) +(Prev2*(1-Se_PCR)))

PPV_Screen_Prev2 <- (Prev2*Se_Screen) / (((1-Prev2)*(1-Sp_Screen)) + (Prev2*Se_Screen))

NPV_Screen_Prev2 <- (Sp_Screen*(1-Prev2))/((Sp_Screen*(1-Prev2))+(Prev2*(1-Se_Screen)))

#=== COMPUTING DIAGNOSTIC ACCURACY WHEN TESTS ARE INTERPRETED IN SERIES (Ses, Sps) AND PARALLEL (Sep, Spp) GIVEN DEPENDENCE===#

Ses <- Se_PCR*Se_Screen + covp

Sps <- 1-(1-Sp_PCR)*(1-Sp_Screen) - covn

Sep <- 1-(1-Se_PCR)*(1-Se_Screen) - covp

Spp <- Sp_PCR*Sp_Screen + covn

#===COMPUTING PPV AND NPV IN POPULATION 1 AND 2 WHEN PCR AND SCREEN ARE INTERPRETED IN SERIES AND PARALELL GIVEN DEPENDENCE===#

PPVs_Prev1 <- (Prev1*Ses) / (((1-Prev1)*(1-Sps))+(Prev1*Ses))

NPVs_Prev1 <- (Sps*(1-Prev1)) / ((Sps*(1-Prev1))+(Prev1*(1-Ses)))

PPVp_Prev1 <- (Prev1*Sep)/((Prev1*Sep)+((1-Prev1)*(1-Spp)))

NPVp_Prev1 <- (Spp*(1-Prev1))/((Spp*(1-Prev1))+(Prev1*(1-Sep)))

PPVs_Prev2 <- (Prev2*Ses) / (((1-Prev2)*(1-Sps))+(Prev2*Ses))

NPVs_Prev2 <- (Sps*(1-Prev2)) / ((Sps*(1-Prev2))+(Prev2*(1-Ses)))

PPVp_Prev2 <- (Prev2*Sep)/((Prev2*Sep)+((1-Prev2)*(1-Spp)))

NPVp_Prev2 <- (Spp*(1-Prev2)) / ((Spp*(1-Prev2))+(Prev2*(1-Sep)))

}")
